# Chronic Diseases Related to Diet and/or Nutrition in Patients With an Ileostomy – A Scoping Review

**DOI:** 10.1111/jhn.70109

**Published:** 2025-08-28

**Authors:** Tjun Wei Leow, Georgia Herbert, Alexandra Mitchell, Rachel Perry, David J. Humes, Gabrielle Thorpe, Scott Clifford, Charlotte Atkinson, Clare England

**Affiliations:** ^1^ Division of Diabetes, Endocrinology and Gastroenterology, Faculty of Biology, Medicine & Health, University of Manchester Manchester Academic Health Science Centre Manchester UK; ^2^ Gastrointestinal Surgery, NIHR Nottingham Biomedical Research Centre Nottingham University Hospitals NHS Trust and the University of Nottingham, School of Medicine, Queen's Medical Centre Nottingham UK; ^3^ NIHR Bristol Biomedical Research Centre University Hospitals Bristol and Weston NHS Foundation Trust and University of Bristol Bristol UK; ^4^ Adult Social Care North Somerset Council Somerset UK; ^5^ School of Healthcare Sciences Cardiff University Cardiff UK; ^6^ The National Institute for Health and Care Research Applied Research Collaboration West (NIHR ARC West) at University Hospitals Bristol and Weston NHS Foundation Trust Bristol UK; ^7^ School of Health Sciences University of East Anglia Norwich UK

**Keywords:** chronic disease, diet, ileostomy, nutrition, scoping review

## Abstract

**Aim:**

Ileostomy formation alters bowel function and is associated with changes that could lead to the development of chronic diseases. The literature on these diseases has not previously been summarised and specific guidelines on their management are absent. This scoping review aimed to explore the extent and type of literature examining such diseases.

**Methods:**

MEDLINE, Embase, CINAHL, AMED, and Web of Science were searched from database inception to 28 April 2025. Studies on chronic diseases (kidney, bone, metabolic disease, and anaemia) related to diet and/or nutrition in adults with any type of ileostomy were included. Patients on parenteral/enteral nutrition were excluded. Screening and data extraction were conducted by pairs of independent reviewers. Results were reported according to PRISMA‐ScR guidelines.

**Results:**

Twenty independent studies (21 full texts) met the inclusion criteria, with sample sizes ranging from 14 to 19,889. Eight full texts reported on kidney disease, five on anaemia/B12 deficiency, four on bone and metabolic disease respectively. The incidence of CKD ranged from 0% to 63.8%, osteopenia from 29.4% to 48.0%, osteoporosis from 5% to 12%, and metabolic disease from 11.8% to 28%. Anaemia incidence was found to be 7.2% and B12 deficiency ranged from no association to 31.8%.

**Conclusion:**

Current evidence on the risk of chronic diseases associated with ileostomy formation is weakened by studies of small sample size and high heterogeneity of population, methodologies and outcomes. Further studies need to stratify rates by ileostomy indication, length of time with an ileostomy, and effect of ileostomy reversal.

## Summary

- This is the first scoping review to systematically search and incorporate the literature on chronic diseases relating to ileostomy.
- There are associations between ileostomy formation and certain chronic diseases (kidney, bone, metabolic disease and anaemia) but the evidence is limited.
- More robust studies are required to better inform stakeholders about developing nutritional guidelines to improve patient outcomes.

## 1 Introduction

More than 100,000 people in the UK are living with a stoma [1] and it has been estimated that over 7000 new ileostomies are formed annually in England [2]. An ileostomy is indicated in the surgical management of Colorectal Cancer (CRC), Inflammatory Bowel Disease (IBD), and other benign conditions such as infective colitis or trauma where bowel contents are diverted to protect distal anastomoses or act as an alternative evacuation route for bowel content [3, 4].

The creation of an ileostomy alters bowel function, potentially conferring complications such as high output stoma, electrolyte imbalance, and malabsorption [5]. This can lead to secondary complications which include chronic kidney disease (CKD), anaemia, and osteoporosis [6, 7, 8]. A recent observational study reports a fourfold increased risk of CKD following ileostomy formation compared to those without ileostomy [9].

The risk of these complications can be significantly reduced through nutritional supplementation or improved/longer‐term dietary advice [7, 10, 11] however, there are no guidelines on dietary/nutritional management for people with an ileostomy [11, 12]. This is likely to be partly attributed to the paucity of evidence on the incidence of chronic diseases associated with ileostomy formation.

Understanding the significance and burden of increased disease risk secondary to ileostomy formation is vital in providing evidence‐based guidelines. A scoping review is useful to map the literature on evolving or emerging topics and assess knowledge gaps [13]. No existing scoping work in this area of research has been published. Therefore, we have conducted this scoping review to explore the extent and type of literature examining chronic diseases related to diet and/or nutrition in patients with an ileostomy.

## 2 Methods

The scoping review was conducted in accordance with the JBI methodology for scoping reviews and reported according to the Preferred Reporting Items for Systematic Reviews and Meta‐analyses extension for scoping review (PRISMA‐ScR) [14, 15]. A protocol was developed and registered on Open Science Framework (https://doi.org/10.17605/OSF.IO/QGE7T). In line with scoping review guidelines, risk of bias and quality assessment were not conducted [15].

### 2.1 Inclusion Criteria

Inclusion criteria were defined in terms of Population, Concept and Context [15].

#### 2.1.1 Population

Studies that reported chronic diseases related to diet and/or nutrition from any type of ileostomy (permanent, temporary, those that have been reversed, continent ileostomies [Kock pouch]) in adults (≥ 18 years) were included. Exclusion criteria were patients with an ileostomy requiring enteral or parental nutrition or those with restorative proctocolectomy and ileal pouch‐anal anastomosis as the focus was not on the ileostomy.

#### 2.1.2 Concept

The concept for this review was diet or nutrition‐related chronic conditions in patients with any type of ileostomy. Our search focused on diseases related to kidney, bone, metabolic disease, and anaemia due to clear mechanisms for association [8, 16, 17, 18]. Chronic was defined as a health problem that requires ongoing management over years or decades and cannot currently be cured but can be controlled with the use of medication and/or other therapies [19].

Outcomes of interest are listed within the following broad categories:

- Kidney disease: Chronic kidney disease/injury or renal failure.
- Bone disease: Osteoporosis and osteopenia (including low bone density; bone mineral density; bone mineral content); osteoporotic fractures of the wrist, spine, and hip.
- Cardiovascular and metabolic disease: Obesity; type 2 diabetes; dyslipidaemia/hypercholesterolaemia (blood lipid profile/cholesterol (including LDL; HDL; total cholesterol; triglycerides); Chronic hypotension (systolic BP < 100 mmHg in women and < 110 mmHg in men)/hypertension (> 140/90 or on one or more drugs to control blood pressure); Cardiovascular disease (including myocardial infarction; heart attack; stroke; cerebrovascular event; cerebrovascular accident; peripheral artery disease; peripheral vascular disease; venous thromboembolism); gallbladder disease; nonalcoholic fatty liver disease.
- Anaemia: Vitamin B12 and iron.

### 2.2 Context

Evidence for inclusion in this review were community and hospital‐based studies from any country and in any language. No restriction was placed on a date to enable the full extent of the evidence available to be mapped. Studies that only considered dietary intakes (with no outcome condition) were excluded.

### 2.3 Types of Sources

All types of original research (randomised controlled trials (RCTs), nonrandomized controlled trials, before and after studies, interrupted time‐series studies) and observational studies (cohort studies, case‐control studies, cross‐sectional studies, descriptive studies, guidelines) were included. Additionally, trial registers, review articles and conference abstracts were also included. Qualitative studies and opinion pieces were excluded as they did not address the association of nutrition or diet‐related chronic diseases with an ileostomy.

### 2.4 Search Strategy

A systematic search of Embase Classic + Embase, MEDLINE and PreMEDLINEAllied and Complementary Medicine (AMED) (all via OVID), Cumulated Index to Nursing and Allied Health Literature (CINAHL) (via EBSCO), Science Citation Index Expanded (SCIEXPANDED), Cochrane Database of Systematic Reviews, International Standard Randomised Controlled Trial Number (ISRCTN), Clinical‐Trials.gov and Cochrane Central Register of Controlled Trials was conducted from database inception to 28 April 2025 with no language restrictions (see search strategy at Supplementary File S1). These search terms were updated for each database, in accordance with their specific requirements. Reference lists of all included articles and relevant reviews were hand‐searched. Grey literature was identified through the OpenGrey database using Web of Science and Conference Proceedings Citation Index‐Science platform.

### 2.5 Study Selection

Search results from all databases were imported into EndNote v.X9 (Clarivate Analytics, PA, USA) and duplicates removed. Titles, abstracts, and full texts were screened against inclusion/exclusion criteria by pairs of independent reviewers (combination of GH, TWL, AM, CA, RP, CE) using Rayyan (Rayyan Systems Inc) [20]. Any disagreements between the reviewers at each stage of the selection process were resolved through discussion, or with an additional reviewer.

### 2.6 Data Extraction

Data was extracted from articles and documents into a charting form by two independent reviewers. Data extracted included: author(s); year of publication; setting; type of publication; country of origin; type of evidence; study design; population (e.g., age, sex, socioeconomic status); indication for ileostomy; type of ileostomy; co‐morbidities; outcome (in relation to the chronic condition). The charting form was tested initially by two independent reviewers on three articles to check that all relevant information relating to the review questions was extracted. The charting form continued to be adapted as required during the review process for example, medication/medical diet and data collection timeframe were added. Authors of included articles were contacted for clarification of information when necessary (Supplementary File S2).

## 3 Results

**Figure 1:**
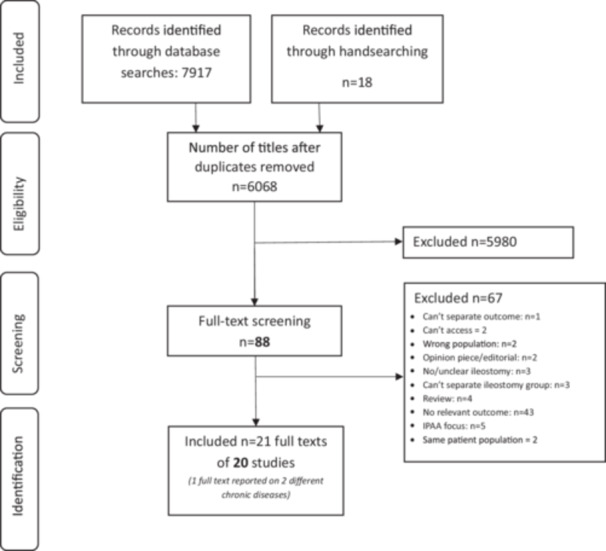
PRISMA‐ScR flow diagram.

The database search retrieved 7917 records, and 18 additional records were identified through handsearching. After removal of duplicates, 6068 titles and abstracts were reviewed. 88 full texts were reviewed of which 21 full texts (20 studies) met the inclusion criteria (Figure 1, Supplementary File S3). Three of the included studies [21, 22, 23] were case reports which provided minimal details and thus not used for evidence synthesis. Details of included full texts are summarised in Table S1. Characteristics of the case reports are summarised in supplementary File S4. There were no registered/ongoing trials relevant to this review at the time of the search.

### 3.1 Characteristics of Included Studies

The 20 included studies were published between 1981 and 2024, including a total sample size of 27,728 (range in cross‐sectional studies: 17–783; range in observational studies 14‐19889). These were undertaken across a range of geographical settings: the majority were conducted in the UK (*n* = 6) [24, 25, 26, 27, 28, 29]. Other countries included USA (*n* = 2) [30, 31], Canada (*n* = 1) [9], Australia (*n* = 2) [21, 22], Finland (*n* = 2) [32, 33], Sweden (*n* = 2) [34, 35], Singapore (*n* = 1) [36], Germany (*n* = 1) [37], Brazil (*n* = 1) [38], Japan (*n* = 1) [39] and not reported (*n* = 1) [23]. Half the studies were retrospective cohort studies (*n* = 10) [9, 24, 25, 27, 28, 30, 31, 34, 36, 39]. The remaining studies were cross‐sectional (*n* = 5) [26, 32, 33, 37, 38], case studies (*n* = 3) [21, 22, 23] or prospective cohort studies (*n* = 2) [29, 35].

Studies within each of the four categories of chronic diseases were identified: kidney disease, bone disease, cardiovascular and metabolic disease, and anaemia. The most explored chronic disease related to diet and/or nutrition in people with an ileostomy was kidney disease (*n* = 8) [9, 24, 25, 26, 30, 35, 36, 39] followed by anaemia/B12 deficiency (*n* = 5) [28, 29, 32, 34, 37], bone disease (*n =* 4) [23, 27, 31, 33] and metabolic disease (with/without CKD) (*n =* 4) [21, 22, 26, 38]. Two studies on metabolic disease [21, 22] and one on bone disease [23] were case studies providing minimal information on the relationship between ileostomy and the chronic condition hence were not discussed further (Supplementary File S4).

### 3.2 Kidney Disease

Eight studies [9, 24, 25, 26, 30, 35, 36, 39], of which four were abstracts [24, 26, 36, 39], examined kidney disease in adults with an ileostomy. All were retrospective cohort studies except for one cross‐sectional [26] and one prospective cohort [35] study. Five studies reported data on sex [9, 25, 26, 30, 35], 5927 (58%) participants were male and 4225 (42%) female. Of those that reported age, the ages ranged from 42 ‐ ≥ 75 yrs. Indications for ileostomy surgery were predominantly colorectal cancer. One study included patients with IBD [9] and another with advanced ovarian cancer requiring debulking surgery [39]. Five studies reported the type of ileostomy that had been created, defunctioning loop [24, 25, 30, 35, 39] and end ileostomy [30]. Six studies [9, 24, 25, 30] provided information on the percentage of ileostomies that had been closed (range: 35%–100%) and five [9, 24, 25, 26, 35] provided timeframes to closure (range: 27 weeks–5 years). There is variation in data collection timeframes with several having collected data at numerous time points (e.g., pre‐ileostomy formation, post‐ileostomy formation, time of reversal) [24, 25, 30]. Incidence of CKD ranges from 0% to 63.8% up to 5 years following ileostomy formation [9, 24, 25, 26, 30, 35, 36, 39] and the risk of CKD was reported as increasing up to five‐fold when compared to those without an ileostomy [9, 30] (Table S1). Smith et al reported a reduced risk of CKD following ileostomy reversal compared to those who did not undergo a reversal (OR –0.97 vs. 2.13) [9]. Additionally, Fielding et al found a significant decrease in eGFR of 4.4 mL/min 6 months postileostomy formation compared to patients without an ileostomy [25] (Table S1).

### 3.3 Cardiovascular and Metabolic Disease

Two studies investigating cardiovascular and metabolic disease were identified [26, 38]. Both were cross‐sectional and one [26] was an abstract. Across both studies, 17 (55%) participants were male and 21 (44%) were female. Indication for ileostomy was reported in one study (cancer and IBD [38]. One study [26] reported percentages of those experiencing hypertension, diabetes mellitus (as well as CKD), and the second study [38] reported proportion of overweight/obese participants post ileostomy formation with no specific timeframe. Reference groups were absent in both studies. The rate of metabolic disease in ileostomates ranged from 11.8% to 38% (Obesity: 11.8% [38], Diabetes Mellitus: 24% [26], Hypertension: 38% [26]).

### 3.4 Bone Disease

Three studies examining bone disease were identified [27, 31, 33], two were retrospective cohorts [27, 31], and one was cross‐sectional [33]. One study [33] compared ileostomy controls with patients who underwent ileal pouch anal anastomosis (IPAA) but, for the purposes of this review, only data from the ileostomy cohort were extracted. Across these studies 88 (43%) participants were male and 118 (57%) were female. All three studies were conducted in participants with IBD [27, 31, 33]. Two studies only included patients without an ileostomy reversal [31, 33], and the proportion of ileostomy closures was not available from the remaining study [27]. Two studies [27, 33] provided information on how long participants had an ileostomy (range 1‐28 yrs), reported details of steroid use and one [31] gave details of other medications that could affect bone health. The main findings were that patients with an ileostomy were reported to have an osteopenia incidence of between 29.4% and 48.0% [27, 31, 33] and an osteoporosis incidence of 5%–12% [27, 33] (Table S1).

### 3.5 Anaemia

One study [28] examining anaemia and five studies [28, 29, 32, 34, 37] investigating B12 deficiency related to diet and/or nutrition in patients with ileostomy were identified: two cross‐sectional [32, 37], two retrospective cohorts [28, 34] and one prospective cohort [29]. Across the studies, the total sample size was 1070 with 57% female and 43% male. Indication for ileostomy was predominantly for IBD and a small proportion was due to familial adenomatous polyposis, indeterminate colitis, or colorectal cancer. The type of ileostomy created included end [29, 37], loop [28], and continent ileostomies [34]. Time to closure was reported in two studies [28]. One study found the incidence of anaemia to be 7.2% [28]. Four studies reported B12 deficiency incidence ranging from 5.1% to 31.2% [28, 29, 34, 37] and one study found no association [32].

## 4 Discussion

### 4.1 Key findings

This scoping review found 21 full texts from 20 studies examining the associations between ileostomy formation and the development of chronic diseases in the domains of kidney, metabolic, bone disease and anaemia.

The incidence of CKD following ileostomy formation is high (up to 63.8%), similar to acute kidney injury (AKI) postileostomy formation [40, 41, 42]. However, contrary to other studies, Rutegard et al [35] did not observe an increased risk of CKD up to 5 years postileostomy formation despite demonstrating positive association of AKI, a predisposing factor to CKD [43]. This is possibly limited by the registry nature of the study. While the decrease in eGFR of 4.4 mL/min in ileostomists seems modest (Table S1), a temporary decline in eGFR has been associated with development of CKD [44, 45]. These findings should be interpreted with caution as CKD aetiology is multifactorial, and most studies did not differentiate ileostomy‐related CKD from other causes such as chemotherapy or IBD [46, 47]. Nonetheless, given that CKD is associated with an increased risk of cardiovascular disease, hospital readmissions, and mortality [48, 49], these findings highlight the potential need for utilisation of strategies such as oral rehydration solution [50] in preventing renal impairment in people with an ileostomy.

Evidence on metabolic and cardiovascular disease in patients with an ileostomy is scarce. Sample sizes were small, reference groups were absent, and causation could not be established due to study designs used. An obesity incidence of 11.8% (Table S1) appears high when several studies have reported lower body weight and BMI in ileostomates [27, 51]. Moreover, changes to gut microbiota (altered by bowel surgery) are associated with insulin resistance and development of diabetes [52] hence there is the potential for increased risks of metabolic disease in people with an ileostomy. This scoping review has shown that this domain remains an understudied area, but the biological plausibility warrants further investigation of associations.

High incidences of osteopenia (29.4%–48.0%) and osteoporosis (5%–12%) are concerning due to their association with an increased risk of fractures [53]. The British Society of Gastroenterology recommends osteoporosis and fracture monitoring for patients with IBD [54]. Given that ileostomy formation is common in IBD surgical management, it is debatable if this should apply to ileostomates. However, the indication for ileostomy in this group was exclusively IBD, a well‐established risk factor for bone disease [55, 56]. Furthermore, steroid usage, a major contributing factor to bone disease [57] was not accounted for. Therefore, establishing the true rate of ileostomy‐related bone disease remains challenging, but crucial as this can be prevented [58].

Similar to metabolic disease, evidence of anaemia among ileostomates is scarce with only one study on 14 participants [28] and no evidence of iron deficiency. Conversely, the evidence around B12 deficiency is conflicting with mixed results. This is likely attributed to heterogeneity in the definition of B12 deficiency (Table S1), varying ileostomy indications, different ileostomy types, uncertainty if B12 deficiency was present before ileostomy formation and length of time for B12 deficiency to develop following ileostomy formation. Crohn's Disease with terminal ileum involvement is known to cause B12 malabsorption, potentially progressing to megaloblastic anaemia [59] and surgical management of Crohn's disease commonly involves terminal ileal resection with ileostomy formation. This highlights the need to stratify the predisposing factors of B12 deficiency to accurately determine its true association with ileostomy formation.

### 4.2 Strengths and Limitations

This is the first scoping review to systematically search and incorporate the literature on chronic diseases relating to ileostomy. The methods employed to identify the included studies were stringent and systematic which helped to ensure a comprehensive search and reduce bias. Our study design was robust, identifying four domains of clinically important chronic diseases that can significantly affect patient morbidity, mortality, and quality of life. Additionally, our study team comprised a multidisciplinary team of reviewers providing clinical and methodological diversity.

There remain inherent limitations. Firstly, confounding factors of the chronic diseases were not accounted for in most studies and most were comprised of a heterogenous sample population where indications for ileostomy were not separated. People with IBD often receive steroids [60], sections of small bowel are resected in Crohn's disease [61] while neoadjuvant/adjuvant chemo/radiotherapy is utilised to treat CRC [62]. Coupled with the underlying disease pathophysiology, such treatment modalities predispose patients to certain chronic diseases. Stratifying these factors is vital to understand the risks in relation to ileostomy and tailor the management plan for the respective patient groups.

Second, data on type of ileostomy (permanent or temporary) and the proportion of ileostomy reversal were mostly absent. For example, renal impairment was reported to persist among ileostomates 3 months after hospital discharge following AKI‐related readmissions [30] and ileostomy reversal attenuates the risk of long‐term kidney injury [9]. Mapping the risk of chronic diseases in relation to the length of time with an ileostomy and if reversal provides any improvement will deepen our understanding.

Contact with the authors of studies was made to obtain further missing data but no response was received (Supplementary File S2). Presently, the number of studies available is small and there is high heterogeneity in methodologies and outcomes hence a meta‐analysis to examine causal relationships with the current studies would be challenging.

### 4.3 Recommendations for Research

This scoping review has identified considerable gaps in the current research literature and between what has been achieved in research and what is needed. Detailed demographic data collection (steroid usage, indications for ileostomy) in future research is necessary to enable stratification. The effects of ileostomy reversal on the incidence of chronic conditions are vital to improving understanding of causality. Future research will require larger sample sizes to produce reliable results, for example, utilisation of primary and secondary care databases such as Clinical Practice Research Datalink (CPRD) [63] and Hospital Episode Statistics (HES) [64]. Additionally, patient and public involvement is crucial to ensure we design studies that are relevant and practical for patients and produce findings that inform clinical practice.

### 4.4 Conclusion

This scoping review found limited literature reporting on the risk of chronic diseases following ileostomy formation. Most evidence came from cohort or cross‐sectional studies and examined the potential association between ileostomy formation and CKD. More studies that detail the incidence of chronic diseases in people with an ileostomy would help to accurately quantify the risk. Once incidence/association has been established, intervention trials are needed to inform guidelines and improve health outcomes.

## Author Contributions

Georgia Herbert, Alexandra Mitchell, Rachel Perry, David J. Humes, Gabrielle Thorpe, Scott Clifford, Charlotte Atkinson and Clare England contributed to the conceptualisation and development of the study, protocol and search. Rachel Perry wrote and carried out the search. Georgia Herbert, Tjun Wei Leow, Alexandra Mitchell, Rachel Perry, Charlotte Atkinson and Clare England completed data extraction. All authors reviewed, edited and approved the final version of this manuscript.

## Ethics Statement

This is a review paper which does not involve any human or animal subjects, therefore ethics approval is not relevant.

## Conflicts of Interest

The authors report no conflicts of interest. The views expressed in this paper are those of the author(s) and not necessarily those of the NHS, the National Institute for Health Research or the Department of Health.

## 1 Peer Review

The peer review history for this article is available at https://www.webofscience.com/api/gateway/wos/peer-review/10.1111/jhn.70109.

## Supporting information

supplementary_Files.

**Table 1:** Characteristics of included studies that examined chronic diseases related to diet and/or nutrition in people with an ileostomy.

## Data Availability

The data that supports the findings of this study are available in the supporting material of this article.
