# Supplementary material for: Chronic Diseases Related to Diet and/or Nutrition in Patients With an Ileostomy – A Scoping Review
Source: J Hum Nutr Diet. 2025 Aug 28;38(5):e70109. doi: 10.1111/jhn.70109 (PMC12391853; doi:10.1111/jhn.70109)
Supplement: Supplementary file 1 — supplementary_Files. [file JHN-38-0-s001.docx]

### Supplementary File 1: Medline search

**ileostomy Medline only**

1. ileostom*.ti,ab.

2. exp Ileostomy/

3. 1 or 2

4. (renal adj1 condition*).ti,ab.

5. (acute adj1 kidney adj1 injur*).ti,ab.

6. (acute adj1 kidney adj1 disease*).ti,ab.

7. (chronic adj1 kidney adj1 disease*).ti,ab.

8. (renal adj1 failure*).ti,ab.

9. (kidney adj1 stone*).ti,ab.

10. (renal adj1 stone*).ti,ab.

11. cystine.ti,ab.

12. (renal adj1 calcul*).ti,ab.

13. struvite.ti,ab.

14. (uric adj1 acid).ti,ab.

15. (calcium adj1 oxalate).ti,ab.

16. dehydration.ti,ab.

17. (bone adj1 disease*).ti,ab.

18. osteoporosis.ti,ab.

19. osteopenia.ti,ab.

20. (osteoporotic adj1 fracture*).ti,ab.

21. fracture*.ti,ab.

22. exp Osteoporosis/

23. exp Kidney Diseases/

24. (bone adj1 densit*).ti,ab.

25. (bone adj1 mineral adj1 densit*).ti,ab.

26. (micronutrient adj1 deficienc*).ti,ab.

27. (cardiovascular adj1 disease*).ti,ab.

28. (myocardial adj1 infarction*).ti,ab.

29. (heart adj1 attack*).ti,ab.

30. stroke*.ti,ab.

31. (heart adj1 disease*).ti,ab.

32. (cerebrovascular adj1 event*).ti,ab.

33. (cerebrovascular adj1 accident*).ti,ab.

34. (peripheral adj1 artery adj1 disease*).ti,ab.

35. (peripheral adj1 vascular adj1 disease*).ti,ab.

36. (venous adj1 thromboembolism*).ti,ab.

37. exp Cardiovascular Diseases/

38. obesity.ti,ab.

39. obese.ti,ab.

40. diabetes.ti,ab.

41. type 2 diabetes.ti,ab.

42. type II diabetes.ti,ab.

43. pre-diabetes.ti,ab.

44. pre-diabetes.mp. or pre diabetes.ti,ab. [mp=title, abstract, original title, name of substance word, subject heading word, floating sub-heading word, keyword heading word, organism supplementary concept word, protocol supplementary concept word, rare disease supplementary concept word, unique identifier, synonyms]

45. hypertension.ti,ab.

46. (blood adj1 pressure*).ti,ab.

47. dyslipid?mia.ti,ab.

48. hypercholesterol?mia.ti,ab.

49. triglycerides*.ti,ab.

50. (total adj1 cholesterol).ti,ab.

51. ((LDL or HDL) adj1 cholesterol).ti,ab.

52. (high adj1 blood adj1 pressure).ti,ab.

53. (insulin adj2 resistance).ti,ab.

54. (oral adj1 glucose adj1 tolerance*).ti,ab.

55. (impaired adj1 glucose adj1 tolerance*).ti,ab.

56. (impaired adj1 fasting adj1 glucose).ti,ab.

57. (blood adj1 lipid adj1 profile*).ti,ab.

58. (blood adj1 lipid adj1 cholesterol).ti,ab.

59. (metabolic adj1 syndrom*).ti,ab.

60. (blood adj1 pressure*).ti,ab.

61. (weight adj1 loss).ti,ab.

62. (metabolic adj1 syndrom*).ti,ab.

63. "non-alcoholic fatty liver disease".ti,ab.

64. Hypokalemia.ti,ab.

65. Hypomagnesaemia.ti,ab.

66. An?emia.ti,ab.

67. (pernicious adj1 an?emia).ti,ab.

68. (megaloblastic adj1 an?emia).ti,ab.

69. Hyponatr?emia.ti,ab.

70. (electrolyte adj1 (imbalance* or disturbance*)).ti,ab.

71. exp vitamin B12 deficiency/

72. (electrolyte adj1 deficienc*).ti,ab.

73. exp anemia/

74. hypophosphatemia.ti,ab.

75. 4 or 5 or 6 or 7 or 8 or 9 or 10 or 11 or 12 or 13 or 14 or 15 or 16 or 17 or 18 or 19 or 20 or 21 or 22 or 23 or 24 or 25 or 26 or 27 or 28 or 29 or 30 or 31 or 32 or 33 or 34 or 35 or 36 or 37 or 38 or 39 or 40 or 41 or 42 or 43 or 44 or 45 or 46 or 47 or 48 or 49 or 50 or 51 or 52 or 53 or 54 or 55 or 56 or 57 or 58 or 59 or 60 or 61 or 62 or 63 or 64 or 65 or 66 or 67 or 68 or 69 or 70 or 71 or 72 or 73 or 74

76. 3 and 75

77. animals/ not humans/

78. Animals, Laboratory/ or Animals/

79. Models, Animal/

80. exp Rodentia/

81. (mouse or mice).ti,ab.

82. 77 or 78 or 79 or 80 or 81

83. 76 not 82

### Supplementary File 2: Contact with authors

| **Author (year)** | **Contact authors for full text and queries** | **Date and type of contact** | **Reply** |
| --- | --- | --- | --- |
| Bong 2018  [abstract] | Paper showed changes in haemoglobin on a graph – authors contacted for exact numbers | 01/09/2022  Corresponding author is Lim | No response |
| Cuinas (2016) [abstract] | Asked for full text | Contacted through researchgate 24/11/2022 | No response |
| M’Koma (2000) | Paper reported the levels of cholesterol but did not state the number of patients with high cholesterol levels or dyslipidaemia – asked for this information. | 28/07/2022  amkoma@mmc.edu or amosy.e.mkoma@vumc.org | 02/08/22 all had normal serum/plasma cholesterol levels up to 36 months after surgery - exclude |
| Ng (2002) [abstract]; Ng (2013) full text | Write to see if 2002 and 2013 connected and if not is there a full paper for the 2002 abstract | 28/07/2022  c.pither@doctors.org.uk | No response |
| Tan (2021)  [abstract] | Asked for full text | Emailed 29/07/2022  yanyu@doctors.org.uk | Replied 30/07/2022- only abstract – no full text |
| Teo (2021)  [abstract] | Asked for full text | Emailed 15/11/2022  No contact details on abstract. Found third author Winson Jianhong Tan on researchgate – contacted through messaging service. | No response |
| Smith (2017)  [abstract] | Asked for full text | Emailed 29/7/2022  mjames@ucalgary.ca | No response |

### Supplementary File 3: Reasons for excluding full texts

| **No.** | **Authors** | **Year** | **Title** | **Reason for exclusion** |
| --- | --- | --- | --- | --- |
|  | Ng et al. | 2002 | Bone mineral density and mineral status of ileostomy patients | Abstract duplicate of full text |
|  | Smith et al. | 2017 | Ileostomy Formation is Associated with Community-Acquired Acute Kidney Injury and New Onset Chronic Kidney Disease | Abstract duplicate of full text |
|  | Chiplunker et al. | 2018 | ALTERED BODY COMPOSITION IN PATIENTS WITH SHORT BOWEL SYNDROME | Can’t separate ileostomy group |
|  | Bath et al. | 2016 | Outcomes after kidney injury in surgery (OAKS): Protocol for a multicentre, observational cohort study of acute kidney injury following major gastrointestinal and liver surgery | Can’t separate ileostomy group |
|  | Greenstein et al. | 1976 | The extra-intestinal complications of Crohn's disease and ulcerative colitis: a study of 700 patients | Can’t separate ileostomy group |
|  | Calvo et al | 2014 | Renal failure associated with intestinal transplantation: Our experience in Spain | Can’t separate outcome |
|  | Hardouin and Loizeau | 1965 | [Medical sequelae of permanent abdominal ileostomy (with a special mention of postoperative neurologic complications)] | Can't access |
|  | Popesco-Urlueni | 1968 | (Intestinal anastomotic syndrome) | Can't access |
|  | Actrn | 2019 | Efficacy trial of a novel stoma-output recycling device | No relevant outcome |
|  | Anonymous | 1993 | Minerva | No relevant outcome |
|  | Arenas et al. | 2012 | HYPOMAGNESAEMIA SEVERE IN PATIENTS WITH HIGH FLOW ILEOSTOMY | No relevant outcome |
|  | Baker et al. | 2021 | A systematic review and meta-analysis of outcomes after elective surgery for ulcerative colitis | No relevant outcome |
|  | Abudeeb et al. | 2017 | Defunctioning stoma- a prognosticator for leaks in low rectal restorative cancer resection: A retrospective analysis of stoma database | No relevant outcome |
|  | Battersby et al. | 2013 | Renal function remains significantly deranged 3 months after anterior resection when a defunctioning loop ileostomy is used rather than a loop colostomy | No relevant outcome |
|  | Beck-Kaltenbach et al. | 2011 | Renal impairment caused by temporary loop ileostomy | No relevant outcome |
|  | Bong et al | 2018 | Comparison of Anthropometric Parameters after Ultralow Anterior Resection and Abdominoperineal Resection in Very Low-Lying Rectal Cancers | No relevant outcome |
|  | Dayton et al. | 1996 | Should older patients undergo ileal pouch-anal anastomosis? | No relevant outcome |
|  | De Paula et al. | 2018 | Surgical Management of Colovesical Fistula in Complicated Diverticular Disease | No relevant outcome |
|  | Gyde et al. | 1984 | Ulcerative colitis: why is the mortality from cardiovascular disease reduced? | No relevant outcome |
|  | Kennedy et al. | 1983 | Calcium metabolism in subjects living with a permanent ileostomy | No relevant outcome |
|  | M’Koma et al. | 2000 | Observations in the blood lipid profile in patients undergoing restorative proctocolectomy | No relevant outcome |
|  | Pace et al. | 2014 | Skin bridge loop ileostomy: A long lasting single center experience | No relevant outcome |
|  | Nagle et al. | 2012 | Ileostomy pathway virtually eliminates readmissions for dehydration in New Ostomates | No relevant outcome |
|  | Magro et al. | 2017 | Third European evidence-based consensus on diagnosis and management of ulcerative colitis. Part 1: Definitions, diagnosis, extra-intestinal manifestations, pregnancy, cancer surveillance, surgery, and ileo-anal pouch disorders | No relevant outcome |
|  | Mala et al. | 2008 | Morbidity related to the use of a protective stoma in anterior resection for rectal cancer | No relevant outcome |
|  | Manzenreiter et al. | 2018 | A proposal for a tailored approach to diverting ostomy for colorectal anastomosis | No relevant outcome |
|  | Messaris et al. | 2011 | Dehydration leads to frequent readmission after ileostomy creation | No relevant outcome |
|  | Ouro et al. | 2021 | Loop ileostomy in rectal cancer surgery: factors predicting reversal and stoma related morbidity | No relevant outcome |
|  | Ozgur et al. | 2019 | Diverting ileostomy: Reversal ratio and non-reversing reason | No relevant outcome |
|  | Pak et al. | 2017 | Predictors of High-Output Stoma After Low Anterior Resection With Diverting Ileostomy for Rectal Cancer | No relevant outcome |
|  | Park et al. | 2018 | Health economic analysis in a randomized trial of early closure of a temporary ileostomy after rectal resection for cancer (easy trial) | No relevant outcome |
|  | Perez et al. | 2015 | High output stomas and dehydration hospital readmission | No relevant outcome |
|  | Perotti et al. | 2020 | Early and medium-term outcomes of ileal pouch-anal anastomosis with combined transanal and laparoscopic approach for ulcerative colitis: Initial experience | No relevant outcome |
|  | Prassas et al. | 2020 | Loop ileostomy versus loop colostomy as temporary deviation after anterior resection for rectal cancer | No relevant outcome |
|  | Quinn et al. | 2014 | Physiological and pharmacological properties of a modified Brooke ileostomy: Justification for retaining the most distal ileum | No relevant outcome |
|  | Petit et al. | 1999 | [Results and indications of lateral ileostomy functionally terminated in colorectal surgery] | No relevant outcome |
|  | Scarpa et al. | 2008 | Restorative proctocolectomy for ulcerative colitis: impact on lipid metabolism and adipose tissue and serum fatty acids | No relevant outcome |
|  | Schrock et al. | 1979 | Complications of continent ileostomy | No relevant outcome |
|  | Servino et al. | 1979 | Some sequelae of definitive ileostomy and of ileo-rectostomy | No relevant outcome |
|  | Setticarraro et al. | 1994 | The first 10 years experience of restorative proctocolectomy for ulcerative-colitis | No relevant outcome |
|  | Yang et al. | 2021 | Temporary impairment of renal function in patients with rectal cancer treated with diverting ileostomy | No relevant outcome |
|  | Seo et al. | 2018 | Readmissions after Ileostomy Creation Using a Nationwide Database | No relevant outcome |
|  | Sohngen et al. | 2015 | Rehabilitation of Patients with Acid-base and Fluid Balance Disorders with Short Bowel Syndrome after Ileostomies | No relevant outcome |
|  | Khaldi et al | 2023 | Deterioration in renal function after stoma creation: a retrospective review from a Middle Eastern tertiary care center | No relevant outcome |
|  | Assaf et al | 2023 | Predisposing factors for high output stoma in patients with a diverting loop ileostomy after colorectal surgeries | No relevant outcome |
|  | Borucki et al | 2023 | Postoperative decline in renal function after rectal resection and all-cause mortality: a retrospective cohort study | No relevant outcome |
|  | Buchli et al | 2022 | Diuretics and RAAS inhibitors increase the risk of dehydration and renal failure in patients with a temporary ileostomy | No relevant outcome |
|  | Loria et al | 2023 | Major renal morbidity following elective rectal cancer resection by the type of diverting ostomy | No relevant outcome |
|  | Mantle et al | 2020 | Nutritional supplementation for vitamin B12 and vitamin K2 deficiency following ileostomy or colostomy formation | No relevant outcome |
|  | Westfall et al | 2024 | Postoperative Oral Rehydration and Regimented Follow-up Decrease Readmissions After Colorectal Surgery That Includes Ileostomies | No relevant outcome |
|  | Yang et al | 2021 | Temporary impairment of renal function in patients with rectal cancer treated with diverting ileostomy | No relevant outcome |
|  | Abitbol et al. | 1997 | Bone assessment in patients with ileal pouch anal anastomosis for inflammatory bowel disease | no/unclear ileostomy |
|  | Hakala et al. | 1997 | Impaired absorption of cholesterol and bile acids in patients with an ileoanal anastomosis | No/unclear ileostomy |
|  | Matar et al. | 2017 | Analysis of the outcome of routine covering ileostomy in laparoscopic and open Total Mesorectal Excision (TME) | No/unclear ileostomy |
|  | Nagle et al. | 2013 | Toward better understanding of readmissions for physiologic complications of ileostomy | Opinion piece/Editorial |
|  | Sekkarie | 2020 | End-stage kidney disease in patients with ileostomies: Challenges and opportunities | Opinion piece/Editorial |
|  | Borucki et al. | 2020 | Risk and consequences of dehydration following colorectal cancer resection with defunctioning ileostomy? A systematic review and meta-analysis | Review |
|  | Borucki et al. | 2021 | Risk and consequences of dehydration following colorectal cancer resection with diverting ileostomy. A systematic review and meta-analysis | Review |
|  | Christl and Scheppach | 1997 | Metabolic consequences of total colectomy | Review |
|  | Gupta et al. | 2013 | Bone loss in patients with the ileostomy and ileal pouch for inflammatory bowel disease | Review |
|  | Contreras et al. | 1976 | Osteomalacia secondary to chronic metabolic acidosis (author's transl) | Wrong population |
|  | Helge | 1963 | [Vitamin B12 deficiency following ileal resection] | Wrong population |
|  | Khanna and Shen | 2012 | Adverse metabolic sequelae following restorative proctocolectomy with an ileal pouch. | IPAA focus |
|  | McLaughlin et al. | 2010 | Osteoporosis in patients over 50 years of age following restorative proctocolectomy for ulcerative colitis: is DXA screening warranted? | IPAA focus |
|  | Navaneethan et al. | 2011 | Influence of ileal pouch anal anastomosis on bone loss in ulcerative colitis patients. | IPAA focus |
|  | Oikonomou et al. | 2007 | Risk factors for anaemia in patients with ileal pouch–anal anastomosis. Dis Colon Rectum 2007; 50:69–74 | IPAA focus |
|  | Shen et al. | 2009 | Risk factors for low bone mass in patients with ulcerative colitis following ileal pouch–anal anastomosis. | IPAA focus |

### Supplementary File 4: Case reports/series

| **Reference (author, year, country) [abstract]** | **Demographics (Age, Sex, Ethnicity)** | **Ileostomy type** | **Indication for ileostomy** | **Past Medical History** | **Medications** | **Data collection timeframe** | **Chronic disease** | **Finding** |
| --- | --- | --- | --- | --- | --- | --- | --- | --- |
| Brunker (2015)  NR  (Abstract) | 83 Female  Ethnicity: N/A | N/A | Ulcerative colitis | Osteoporosis  Osteoarthritis, Psoriasis, Ulcerative Colitis | Bisphosphonate, Guaifenesin, Methotrexate, Hydrocodone, Acetaminophen, Zolpidem | N/A | Bone health | Multiple atraumatic osteoporotic pelvic fractures |
| Clements (2020)  Australia | 57 Female  Ethnicity: N/A | Permanent | Ulcerative colitis | Ulcerative colitis, primary sclerosing cholangitis, cirrhosis | N/A | N/A | Metabolic health | Variceal bleed from ileostomy site due to portal hypertension |
| Cameron (1970)  Australia and New Zealand | 41 Female  Ethnicity N/A  35 Female  Ethnicity N/A | N/A | Ulcerative colitis | Patient 1: Ulcerative Colitis, cirrhosis  Patient 2: Ulcerative colitis, cirrhosis | N/A | N/A | Metabolic health | Both patients: Variceal bleed from ileostomy site due to portal hypertension |
