# Supplementary material for: Chronic Diseases Related to Diet and/or Nutrition in Patients With an Ileostomy – A Scoping Review
Source: J Hum Nutr Diet. 2025 Aug 28;38(5):e70109. doi: 10.1111/jhn.70109 (PMC12391853; doi:10.1111/jhn.70109)
Supplement: Supplementary file 2 — Table 1: Characteristics of included studies that examined chronic diseases related to diet and/or nutrition in people with an ileostomy. [file JHN-38-0-s002.docx]

### Table 1. Characteristics of included studies that examined chronic diseases related to diet and/or nutrition in people with an ileostomy.

| **Source** | | | **Population** | | | | | **Concept** | | | |
| --- | --- | --- | --- | --- | --- | --- | --- | --- | --- | --- | --- |
| **Reference (author, year, country) [abstract]** | **Evidencetype** | **N** | **Characteristics (i.e., Confounding factors)** | | | | | | **Data collection timeframe** | **Chronic disease** | **Main finding N (%)** |
|  |  |  | ***Indication for ileostomy*** | ***Type of ileostomy/surgery (% closed; time to closure)*** | ***Age (y), sex (N,%), ethnicity*** | ***Pre-existing diseases, N (%)*** | ***Medication/medical diets*** | |  |  |  |
| **Kidney Disease: Chronic kidney disease/injury or renal failure** | | | | | | | | | | | |
| Cuinas et al. (2016)  UK  [abstract] | RC | 107 | Rectal cancer | Defunctioning loop ileostomy (100% closed)  Median time to closure: 33wks | NR | NR | NR | | -Pre-op  -Time of reversal -12m post-ileostomy formation | Persistent renal impairment (eGFR<60 ml/min) | Median eGFR  Preop: 86 ml/min (IQR 24.3)  Time of reversal: 72 ml/min (IQR 31.5) (p<0.05)  12m post ileostomy formation: 74 ml/min (IQR 30)  Number of patients with renal impairment (eGFR<60 ml/min) increased at reversal (14.0% vs 28.0%; p<0.05) and didn’t improve by 12m (21.5%)  12 patients (10%) re-admitted for renal failure. |
| Fielding et al. (2020)  UK | RC | 583 | Rectal cancer | Total mesorectal excision and loop ileostomy (% closed: 494/583 (85%); 56 died; 30 not closed by end of study)  Median time to closure: 27wks (mean 32, IQR 17-42). | Median age 68 (IQR 61-74)  M: 396 (67.9%)  F: 187 (32.1%) | NR | NR | | T0: Pre-ileostomy formation  T1: 6 months post-ileostomy formation  T2: 6 months following ileostomy closure | Moderate/  severe CKD (stage 3 & above) | Post op eGFR +/- SD (ml/min/1.73 m^2^)   \|  \| Ileostomy \| No ileostomy \| \| --- \| --- \| --- \| \| T0 \| 78.3 +/- 17.1 \| N/A \| \| T1 \| 73.9 +/- 0.83 \| N/A \|   No significant decline for non-ileostomates  Rates of moderate/severe CKD   \|  \| Ileostomy \| No ileostomy \| \| --- \| --- \| --- \| \| T0: \| 78 (13.4%) \| 119 (19.0%) \| \| T1: \| 129 (23.0%) \| 91 (18.3%) \| \| T2: \| 150 (28.4%) \| 144 (25.9%) \|     Multivariable regression: Ileostomy associated with decline in GFR of 5.3ml/min (p<0.001) |
| Li et al. (2017)  USA | RC | 84  (Total: 619) | Colorectal cancer | End ileostomy: n=28 (33%)  Among those with  diverting ileostomy, 42 (75.0%) underwent reversal (no info on time to reversal) | Age category:  <65: 44 (52.4%)  65-74: 25 (29.8%)  ≥75: 15 (17.9%)  M: 83 (98.8%)  F: 1 (0.2%)  White: 56 (66.7%), Black: 24 (28.6%), Other): 4 (4.7%) | DM:  23 (27.4%)  Peripheral vascular disease: 1 (1.2%)  MI (within 6m): 0 | NR | | 12m post-surgery, and ‘over time’ | Severe CKD | Development of severe CKD at 2 years:  Ileostomy - 25%  No ileostomy - 8%  In univariate analysis, ileostomy was an independent predictor of severe CKD at 1y (OR, 2.9; 95% CI, 1.2-7.0, P = 0.02) and also after adjusting for confounders (OR, 4.1; 95% CI, 1.4-11.9, P < 0.01) |
| Teo et al. (2021)  Singapore  [Abstract] | RC | Ileostomy: 246 (No-ileostomy: 246) | Rectal cancer | NR | NR | NR | NR | | 2003-2013 | CKD | New-onset CKD and/or progression of their pre-existing CKD: 24%  Newly initiated late RRT: 2.5% |
| Smith et al. (2021)  Canada | RC | Ileostomy: 4136  No-ileostomy: 15,753 | IBD, colon, rectal or rectosigmoid cancer | Reversal within 1y of discharge: 1464 | Mean age: 57.8 (SD 16.0)  M: 2332 (56.4%)  F: 1804 (43.6%) | CVD: 85 (2.1%) HF: 130 (3.1%): Chronic pulmonary disease: 369 (8.9%): DM: 493 (12.0%); MI: 205 (5.0%) Liver disease: 109 (2.7%); Peripheral vascular disease 163 (3.9%) | NR | | Within 1 year of discharge | CKD | New onset CKD: 216/3383 (6.4%)  New-onset CKD among patients without  previous community-onset AKI: Ileostomy: 80/2906 (2.8%)  Unadjusted OR (95% CI) 1.74 (1.33–2.26) Adjusted OR (95% CI) 2.45 (1.85-3.23)  New-onset CKD among patients with  previous community-onset AKI: Ileostomy: 136/477 (28.5%)  Unadjusted OR (95% CI) 3.09 (2.21–4.34)  Adjusted OR (95% CI) 4.99 (3.42-7.28)  New-onset CKD no reversal vs reversal:  Adjusted OR 2.13 (95% CI: 1.63–2.79) vs 0.97 (95% CI: 0.67–1.40)  New-onset CKD among patients with previous community-onset AKI (no reversal vs reversal):  Adjusted OR 4.47 (95% CI: 2.67– 7.49) vs 2.49 (95% CI: 1.50–4.12) |
| Rutegard et al. (2023)  Sweden | PC | 5355  Ileostomy: 4364  No ileostomy:  991 | Rectal cancer | Defunctioning loop ileostomy  Stoma reversal:  3494/4364 (80.1%)  Reversal within 90 days: 331 (9.5%)  Reversal after 90 days: 3163 (90.5%) | Median age:  66  (IQR 59-72)  M: 3128 (58.4%)  F: 2227 (41.6%) | HTN: 1770 (33.1%)  CVD: 446 (8.3%)  HF: 127 (2.4%)  DM: 543 (10.1%)  COPD: 140 (2.6%) | NR | | Post ileostomy formation:  1 year  3 year  5 year | CKD | \|  \| **HR** \| **95% CI** \| \| --- \| --- \| --- \| \| 1 year \| 2.47 \| 0.90-6.81 \| \| 3 year \| 1.76 \| 0.54-5.73 \| \| 5 year \| 0.73 \| 0.34-1.53 \|   No significant association between ileostomy and CKD |
| Fujiwara et al. (2024)  Japan  (Abstract) | RC | 132  Ileostomy:  66  No Ileostomy:  66 | Debulking surgery for advanced ovarian cancer | Diverting ileostomy  Ileostomy reversal: 35/66 (53%) | NR | NR | NR | | NR | CKD | \| **Ileostomy** \| **No Ileostomy** \| \| --- \| --- \| \| 63.8% \| 30.2% \| |
| **Metabolic Disease (with/without CKD)** | | | | | | | | | | | |
| Tan et al. (2021)  UK  [Abstract] | Cross-sectional study | 21 | NR | Median (IQR) length of time of ileostomy: 5y (2-12) | Median age: 63 (IQR 51-73)  M: 15 (71.4%)  Female: 6 (28.6%) | NR | NR | | January 2020 | Cardiovascular and metabolic syndrome (HTN; DM)  CKD | HTN (38%)  DM (24%)  CKD (14%) |
| Moraes et al. (2019)  Brazil | Cross- sectional study | 17 | Cancer, IBD | End ileostomy | Mean age: 55.8 (range 26-80)  M: 6 (35.3%)  F: 11 (64.7%) | DM: 4 (23.5%)  HTN: 3 (17.6%)  Others: 4 (23.5%) | NR | | Time since ileostomy formation.  Mean: 3.25y (1.5m-15y)  <2y: 12 (70.6%)  2-5y: 2 (11.8%)  >5y: 3 (17.6%) | Raised BMI | Overweight: 4 (23.5%)  Obese: 2 (11.8%) |
| **Bone Disease** | | | | | | | | | | | |
| Gupta et al. (2014)  USA | RC | 126 (jejunostomy: n=3; colostomy: n=3) | UC: 16 (13.0%) Crohn’s disease: 110 (87.3%) | Patients with ileostomy closure were excluded | Mean age: 34.8 (SD 13.3)  M: 45 (35.7%)  F: 81 (64.2%)  (Menopause: 33 (40.7%))  White: 118 (93.6%)  African American: 5 (3.9%)  Others: 3 (2.3%) | Renal stone: 20 (15.9%)  DM: 8 (6.3%)  Hypothyroidism: 11 (8.7%)  Short bowel syndrome: 22 (17.5%) | Bone-losing medicine: 15 (11.9%)  Daily Vit. D supplement: 59 (46.8%)  Daily calcium supplement: 52 (41.3%)  Oestrogen replacement (females): 18 (14.3%)  Any steroid use: 114 (90.5%) | | No time period – patients identified from notes | Low BMD ^a^ | Median of 6.6y (interquartile range, 2–18.7y)  Low BMD: 37 (29.4%) |
| Kuisma, et al (2002)  Finland | Cross-sectional | 20 | UC  Mean (SD) duration of UC:  4.0y (1.3) | Patients with ileostomy closure were excluded | Mean age: 52.6 (37–68)  M: 8 (40.0%)  F: 12 (60.0%) | N/A | Prednisone Cumulative dose 5.0 (1.4) g  Daily dose 4.5 (1.8) mg | | Time since surgery, years (range):  19.2 (16–28) | Osteopenia (spine and femoral neck) defined as Z score <-1 Severe osteopenia as Z-score <-2 | **Osteopenia**  Spine = 6 (30.0%)  Femoral neck = 2 (10.0%)    **Severe osteopenia or osteoporosis**  5.0% |
| Ng et al (2013)  UK | RC | 60 | UC: 38 (63.3%) Crohn's Disease: 19 (31.7%)  Other: 3 (5.0%) | Ileostomy formation with SBR/NSBR  SBR: 14 (23.3%)  NSBR: 46 (76.7%) | Mean age: 55.0 (range 25-87)  M: 35 (58.3%)  F: 25 (41.7%) | NR | NR | | Median time post ileostomy formation:  NSBR: 49m (12-405)  SBR: 84.5m  (51-252) | Osteopenia^b^  Osteoporosis^b^ | Osteopenia: 29 (49.1%)  Osteoporosis: 7 (11.9%) |
| **Anaemia** | | | | | | | | | | | |
| Nicholls et al. (1981)  UK | RC | 14 | UC: 10 (71.4%)  FAP: 4 (28.6%) | Loop ileostomy; proctocolectomy with ileal reservoir  (Mean time to closure: 17.2m (Range: 6-28) | Mean age: 31.3 (range 16-49)  M: 7 (50.0%)  F: 7 (50.0%) | NR | NR | | Follow up period taken to start from date of closure | Anaemia,  B12 deficiency | Anaemia: 1 (7.2%)  B12 deficiency: 1 (7.2%) |
| Jayaprakash et al. (2004)  UK | PC | 39 | UC: 17 (43.6%)  Crohn’s disease: 18 (46.2%)  Indeterminate colitis: 4 (10.3%) | End ileostomy | Mean age: 47.0 (range 24.9–73.7)  M: 21 (53.8%)  F: 18 (46.2%) | NR | NR at time of B12 measurement | | Mean duration since ileostomy formation: 12.5y (2.5-35.1) | Vitamin B12 deficiency (defined as serum B12 < 150 ng/l) | Overall mean vitamin B12 level: 545.7 ng/l (68- >2000)  B12 Deficiency: 2 (5.1%) (1 previously undiagnosed)  No significant correlation between vitamin B12 level and duration of ileostomy (s=−0.1, P>0.5).  Subgroup analysis did not show correlation of ileostomy duration with vitamin B12 levels in Crohn’s (s=0.088, P>0.5) or UC patients (s=−0.19, P>0.2). |
| Kuisma et al. 2001  Finland | Cross sectional | 21 | UC | NR | Mean age:  51.7 (range 37–68)  M: 9 (42.8%)  F: 12 (57.1%) | NR | NR | | Mean (range) time since ileostomy formation:  19.2y (16–28) | Vitamin B12 deficiency  (<170 pmol/L) | Mean B12 absorption:  26.0 ± 1.8 (95% CI 22.2–29.8)    No evidence of Vit B12  deficiency |
| Nilsson et al (1984)  Sweden | RC | 213  (total = 235; 22 excluded) | UC: 170 (79.8%)  Crohn's Disease: 30 (14.1%)  FAP: 13 (6.1%) | Continent ileostomy | Mean age: 41 (range 17-70)  M: 107 (44.8%)  F: 128 (55.2%) | NR | NR | | NR | B12 deficiency  (<130pmol/l)  Borderline B12 levels (130-200pmol/l) | B12 deficiency: 14/213 (6.6%)  Borderline Vit B12: 14/213 (6.6%)  2/14 from the borderline group developed Vit B12 deficiency one year later. |
| Schiergens et al (2017)  Germany | Cross- sectional | 783 | UC: 344 (43.9%)  Crohn’s disease: 300 (38.3%)  Colorectal cancer: 51 (6.5%)  FAP: 48 (6.1%)  Other: 40 (5.1%) | End ileostomy | Median age: 59 (range: 24-94)  M: 324 (41.4%)  F: 459 (58.6%) | Short bowel syndrome: 80 (10.2%)  Chronic kidney insufficiency: 121 (15.5%) | NR | | Median time after ileostomy formation: 18y (range: 2-52) | B12 deficiency | 244 (31.2%) |

Key: BMD = bone mineral density; d = days; COPD = Chronic obstructive pulmonary disease; CVD = cardiovascular disease; DM = diabetes mellitus; F = female; FAP = Familial adenomatous polyposis; HF = Heart Failure, HTN = hypertension; N = Hypertension; IBD = inflammatory bowel disease; M = male; m = months; MI = myocardial infarction; NR = Not reported; NSBR-non small bowel resection; PC = prospective cohort; SBR=small bowel resection: RC = retrospective cohort; RRT = renal replacement therapy; SD = standard deviation; UC = ulcerative colitis; uLAR = Ultra-low anterior resection with temporary ileostomy; wks = weeks; y = years

^a^ Defined as a T-score of lumber spine, total hip, or femoral neck of <-2.5 or less in postmenopausal women and in men 50y of age and older; or a Z-score of -2.0 or lower in women before menopause or men younger than 50y)

^b^ According to WHO definition, osteopenia (-1.0<T score<-2.5), osteoporosis (T score <-2.5)

^c^ Error in abstract: 12 patients is not 10%

*19 out of 22 full texts described in detail as 3 provided minimal info and was excluded from our analysis
